# Targeting ANXA7/LAMP5-mTOR axis attenuates spinal cord injury by inhibiting neuronal apoptosis via enhancing autophagy in mice

**DOI:** 10.1038/s41420-023-01612-w

**Published:** 2023-08-24

**Authors:** Na Li, Lu Chen, Xiaoqing Zhao, Chi Gu, Yong Chang, Shiqing Feng

**Affiliations:** 1 Orthopaedic Research Center of Shandong University, Department of orthopaedics, Qilu Hospital of Shandong University, #44 Wenhua West Road, 250012 Jinan, Shandong China; 2 Advanced Medical Research Institute, Shandong University, Jinan, Shandong China

**Keywords:** Apoptosis, Cell death in the nervous system, Autophagy

## Abstract

Spinal cord injury (SCI) could lead to severe disabilities in motor and sensory functions, and cause a heavy burden on patient physiology and psychology due to lack of specific repair measures so far. ANXA7 is an annexin with Ca^2+^ -dependent GTPase activity, which were mainly expressed in neuron in spinal cord and downregulated significantly after SCI in mice. In our study, GTPase activity activation of ANXA7 plays the protective role in neuron after OGD/R through inhibiting neuron apoptosis, which mediated by enhancing autophagy via mTOR/TFEB pathway. We also discovered that ANXA7 has significant interaction with neural-specific lysosomal-associated membrane protein LAMP5, which together with ANXA7 regulates autophagy and apoptosis. Asp411 mutation of ANXA7 obviously impaired the interaction of ANXA7 and LAMP5 compared with the wild type. Furthermore, it was found that activation of ANXA7 could help to stabilize the protein expression of LAMP5. Overexpression of LAMP5 could attenuate the destruction of lysosomal acidic environment, inhibition of autophagy and activation of apoptosis caused by ANXA7 downregulation after OGD/R. We verified that injecting ANXA7 overexpression lentivirus and activation of ANXA7 both have significant repair effects on SCI mice by using CatWalk assay and immunohistochemistry staining. In summary, our findings clarify the new role of ANXA7 and LAMP5 in SCI, provided a new specific target of neuronal repair and discovered new molecular mechanisms of ANXA7 to regulate autophagy and apoptosis. Targeting ANXA7 may be a prospective therapeutic strategy for SCI in future.

## Introduction

Spinal cord injury (SCI) is a serious traumatic disease with high morbidity and disability ratio [1]. It causes impairment of neurological functions such as sensation and movement, and could even lead to temporary or permanent disability [2, 3**]**. Due to the lack of effective treatment strategies, less than 1% of patients suffered from spinal cord injury are discharged with complete neurological recovery [4]. However, there is still a vacancy in exploring more effective repair mechanism in neurons injury process. Therefore, it’s necessary to focus on exploring the molecular mechanism of neuronal injury after spinal cord injury and proposing corresponding solutions in the field of neural repair.

Annexin A7 (ANXA7) is a member of the Annexin family, which binds to calcium ions and phospholipids to exert Ca^2+^-activated GTPase activity [5, 6**]**. ANXA7 regulates autophagy in cardiovascular disease and cancer through regulating cell cycle, drug resistance, autophagy and apoptosis [7–10]. Moreover, studies showed ANXA7 could regulate platelet metabolism and Ca^2+^ release in arterial thrombosis [11]. But the regulatory mechanism of ANXA7 in spinal cord injury is still unclear. Few studies have focused on the regulatory effects of ANXA7 in nerve system. A study showed the crucial role of ANXA7 in secondary brain injury mediated by its phosphorylation after experimental intracerebral hemorrhage in rats [12]. Other studies have mentioned that ANXA7 is significantly upregulated and has a regulatory effect on epilepsy [13]. However, the changes of ANXA7 after spinal cord injury and its regulatory effects on spinal cord injury remain unclear.

Lysosome-associated membrane proteins (LAMPs) are a kind of highly glycosylated protein located on the extracorporeal membrane of lysosomes, which plays a vital role in regulating the function of lysosomes [14]. The lysosomal membrane proteins LAMP1 and LAMP2 are estimated to contribute about 50% of all proteins on the lysosome membrane, which play essential role in lysosome biogenesis and autophagy [15]. As for LAMP5, it is a brain and nerve specific member of the lysosomal associated membrane protein family [16]. But the role of LAMP5 in regulating spinal cord injury is rarely reported. Our previous RNA sequence results showed that LAMP5 was down-regulated most significantly among LAMPs after spinal cord injury, so we guess LAMP5 may have important role in spinal cord injury, which needs to be further explored.

Autophagy is a stress-buffering step of intracellular and extracellular stress. Autophagy inhibition renders cells unable to cope with stressful situations and leads to apoptosis [17**–**20]. Appropriate autophagy activation could reduce inflammation and inhibit apoptosis [21**–**23]. Previous studies have shown that autophagy is inhibited after spinal cord injury, thereby inducing cell death of injured neurons and the deterioration of spinal cord injury. Autophagy activators can significantly improve this situation and prevent neuron death [24**–**26], and AMPK/mTOR/TFEB pathway is involved in this process and plays important roles [27, 28]. As a key pathway in autophagy regulation, inhibition of mTOR pathway after spinal cord injury could be beneficial to spinal cord injury [29, 30]. However, the autophagy regulation mechanism during spinal cord injury is still need to be explored and potential clinical treatment may be put up based on it. In this study, we aimed to explore the new target ANXA7 and corresponding regulatory mechanism that specifically regulate autophagy process and further affect apoptosis in neuron after spinal cord injury.

## Results

### ANXA7 was much more distributed in neurons than microglia, astrocytes and oligodendrocytes in spinal cord of mice

**Fig. 1:**
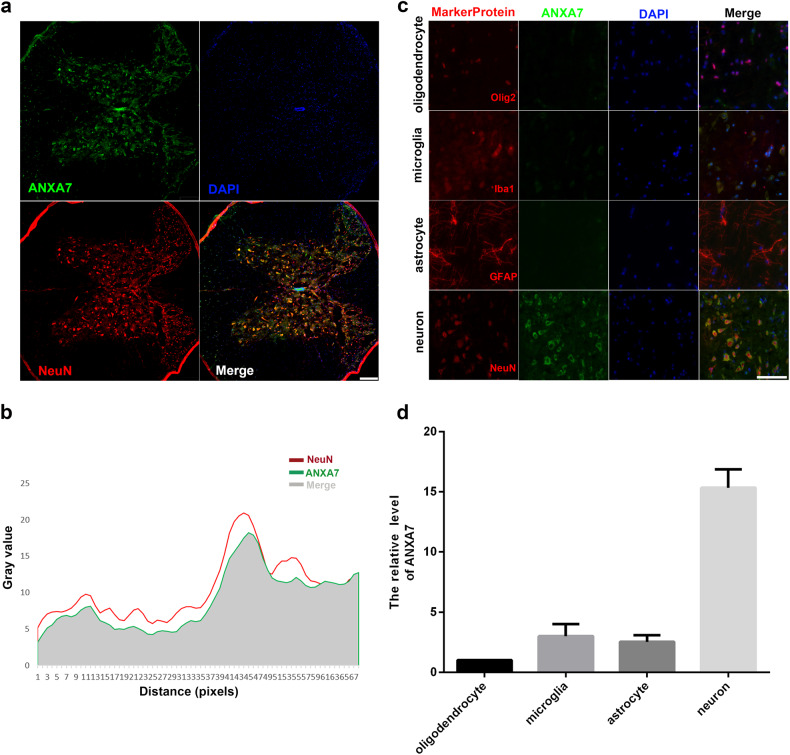
ANXA7 was much more abundant in neurons than microglia, astrocytes and oligodendrocytes in spinal cord of mice. **a** Distribution of ANXA7 and NeuN in spinal cord. Scale bars: 200 µm. **b** ImageJ analysis of co-localization of ANXA7 and NeuN. **c**, **d** Protein level of AXNA7 of astrocyte, microglia, oligodendrocyte and neurons. Scale bars: 100 µm.

In order to explore the distribution of ANXA7 in mouse spinal cord, mouse spinal cord tissues were extracted to execute immunohistochemistry staining. The results showed that ANXA7 was abundant in neurons of spinal cord (Fig. 1a, b). In addition, based on the result, we speculate that ANXA7 specificity is present in neurons and less commonly in other cells of the spinal cord. To verify the hypothesis, we compared the expression of ANXA7 in astrocyte, microglia, oligodendrocyte and neuron in mouse spinal cord tissue, and it’s not hard to see that ANXA7 is much more abundant in neurons than other kinds of cells in spinal cord (Fig. 1c, d). The above results suggested that ANXA7 may have specific regulatory effect on neurons.

### ANXA7 is significantly downregulated after spinal cord injury in mice

**Fig. 2:**
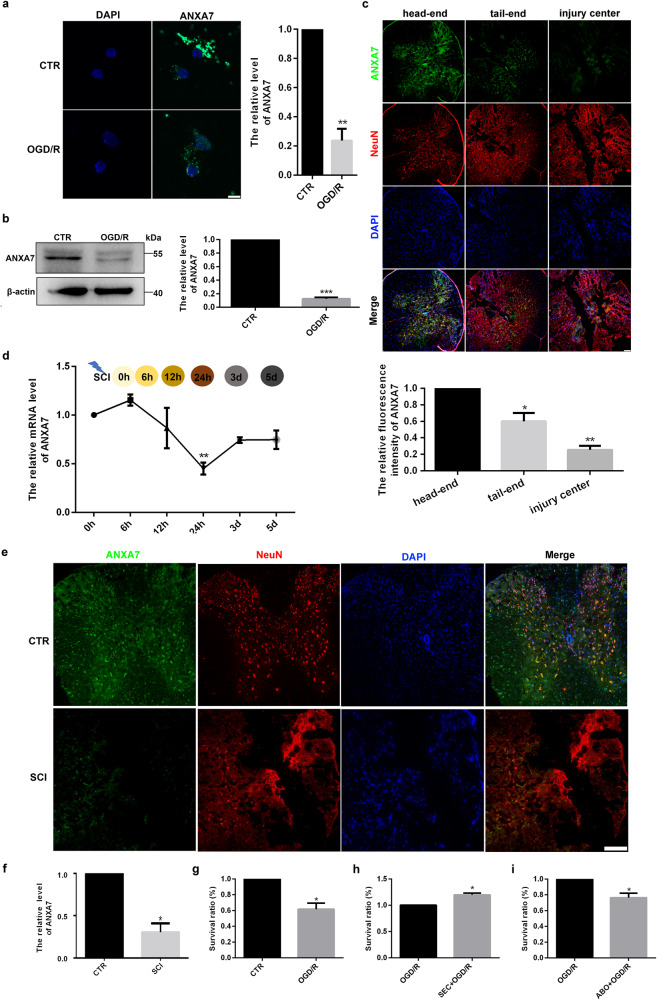
ANXA7 is significantly downregulated after neuron injury. **a** Neurons were treated with OGD/R, after which immunofluorescence staining was used to detect ANXA7 protein level. Scale bars: 10 µm. **b** Neurons were treated with OGD/R and Western blotting was used for protein detection. **c** Spinal cord injury model was performed in C57BL/6 mice, the distribution of ANXA7 were detected by immunohistochemistry staining in head-end, tail-end and injury center in spinal cord after 24 h of injury. Scale bars: 200 µm. **d** ANXA7 mRNA level in mice after 0 h, 6 h, 12 h, 24 h, 3 d and 5 d of spinal cord injury. **e**, **f** Protein level of ANXA7 in neuron after 24 h of spinal cord injury detected by immunohistochemistry staining. **g**–**i** Neurons were treated with 0.1% DMSO, SEC or ABO separately 1 h before OGD/R treatment, and CCK-8 assay was used to detect survival ratio of neurons. Data are presented as means ± SD, * *P* < 0.05, ** *P* < 0.01, *** *P* < 0.001, *n* = 3.

To explore the effect of ANXA7 on spinal cord injury, Oxygen–Glucose Deprivation/Reoxygenation(OGD/R) model of neuron were also conducted, which is considered as the cell model for spinal cord injury [31]. Immunofluorescence assay and Western blotting detection displayed that ANXA7 was down-regulated after OGD/R treatment (Fig. 2a, b). Furthermore, to explore the relationship between ANXA7 expression and injury degree, SCI models were conducted and ANXA7 expression was detected in different area after 24 h of injury. Indeed, the expression of ANXA7 is downregulated following with injury deepens. The expression level of ANXA7 in the injury center was significantly lower than that of the head and tail ends of the injury area (Fig. 2c). To reflect the change of ANXA7 expression more accurately, the mouse spinal cord injury model was constructed and mRNA levels of ANXA7 in a time gradient after spinal cord injury were examined. The results showed that ANXA7 mRNA was significantly downregulated after spinal cord injury, with the lowest expression at the time point 24 h after spinal cord injury (Fig. 2d). Besides, we also examined changes of ANXA7 expression in spinal cord tissues between mice with spinal cord injury and normal controls. The results of immunohistochemistry showed that ANXA7 expression was significantly downregulated in mice after spinal cord injury (Fig. 2e, f). Then, in order to explore the potential protective effect of ANXA7 on neurons after OGD/R, CCK-8 was used to detect the effect of ANXA7 activator SEC, and ANXA7 inhibitor ABO on neuronal survival, respectively. The results showed that OGD/R treatment could decrease survival ratio of neurons, and ANXA7 activation has a protective effect on neurons survival while ANXA7 inhibition has the opposite effect (Fig. 2g–i). Above results suggest that ANXA7 may have the essential regulatory role on spinal cord injury regulation.

### Activation of ANXA7 promotes autophagy and inhibits apoptosis in injured neurons

**Fig. 3:**
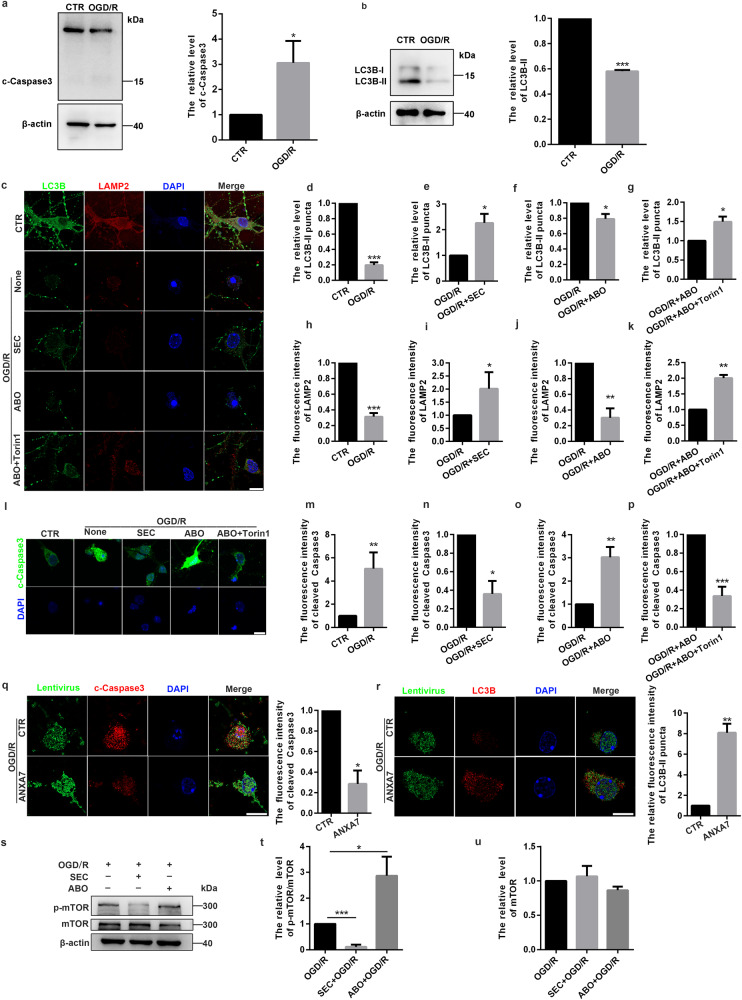
Activation of ANXA7 promotes autophagy and inhibit apoptosis of injured neuron. **a** Western blotting was used to detect protein level of Caspase3 after OGD/R treatment. **b** Western blotting was used to detect protein level of LC3B-II after OGD/R treatment. **c**–**k** Neurons were severally treated with DMSO (0.1%), SEC, ABO or ABO+Torin1 for 1 h before OGD/R treatment, after which immunofluorescence staining was used to detect expression level of LC3B-II and LAMP2. Scale bars: 10 µm. **l**–**p** Neurons were severally treated with DMSO (0.1%), SEC, ABO or ABO+Torin1 for 1 h before OGD/R treatment, after which immunofluorescence staining was used to detect expression level of c-Caspase3. Scale bars: 10 µm. **q**, **r** Neurons were transfected with ANXA7 overexpression lentivirus before OGD/R treatment, after which immunofluorescence was used to detect expression of c-Caspase3 and LC3B-II. Scale bars: 10 µm. **s**–**u** Neurons were severally treated with DMSO (0.1%), SEC and ABO for 1 h before OGD/R treatment, after which Western blotting was used to detect protein level of p-mTOR and mTOR. Data are presented as means ± SD, * *P* < 0.05, ** *P* < 0.01, *** *P* < 0.001, *n* = 3.

It has been reported that OGD/R treatment could cause neurons injury and death. Autophagy activation is an effective way to promote cell survival under stress conditions and LC3B is widely used as the marker of autophagy. When autophagy is activated, LC3B-I (localized to the cytoplasm) is covalently linked to phosphatidyl ethanolamine (PE) and inserted into the bilayer membrane of the autophagosome to form LC3B-II. To verify the effect of OGD/R on neurons, protein levels of LC3B and apoptosis associated protein caspase3 were detected. Western blotting proved that OGD/R treatment could increase expression of cleaved Caspase3 and inhibit expression of LC3B-II (Fig. 3a, b), which indicated that OGD/R treatment can inhibit autophagy and promote apoptosis in neurons. Previous studies have reported that ANXA7 was related with cell survival in prostate cancer, glioma and pancreatic cancer [9, 32]. In order to verify that ANXA7 could also promote cell survival in neurons after OGD/R, neurons were treated with SEC or ABO 1 h before OGD/R treatment. OGD/R treatment could notably inhibit the expression of LC3B-II and autophagy associated protein LAMP2 (Fig. 3c, d, h). We also found that activation of ANXA7 can increase puncta of LC3B-II and the expression of LAMP2 (Fig. 3c, e, i), and inhibition of ANXA7 suppressed the expression of LC3B-II and LAMP2 (Fig. 3c, f, j), activation GTPase activity of ANXA7 could promote autophagy while inhibitor has the opposite effect. ANXA7 can promote neuron survival after spinal cord injury by promoting autophagy. In addition, mTOR suppression by Torin 1 can reverse the inhibition of autophagy caused by ANXA7 inhibitor ABO, which indicates the activation of autophagy by ANXA7 may be mediated through mTOR-dependent manner (Fig. 3c, g, k). Meanwhile, SEC could inhibit neuron apoptosis after OGD/R treatment, while ABO promoted this process, and Torin1 could also rescue neuron apoptosis even treated with OGD/R and ABO (Fig. 3l–p). Furthermore, to verify the protective effect of ANXA7 on injured neurons, ANXA7 overexpression lentivirus was transfected into neurons to upregulate ANXA7 expression. The immunofluorescence results showed that ANXA7 overexpression could reduce the level of c-Caspase3 and increase the level of LC3B, which indicated that ANXA7 could promote autophagy in injured neurons and inhibit apoptosis (Fig. 3q, r). The mTOR signaling pathway is a classic autophagy regulatory pathway, and mTOR inhibition could promote autophagy by promoting the translocation of the transcription factor TFEB into nucleus [33, 34]. To demonstrate the regulatory effect of ANXA7 on mTOR, we examined the effects of SEC and ABO on mTOR in neurons after OGD/R treatment. The results showed that SEC could inhibit the phosphorylation of mTOR, but ABO could promote the phosphorylation of mTOR (Fig. 3s–u). Above results indicated that ANXA7 could activate autophagy and thus inhibit apoptosis via inhibiting activation of mTOR in neurons after OGD/R.

### Activation of ANXA7 promotes autophagy through mTOR/TFEB

**Fig. 4:**
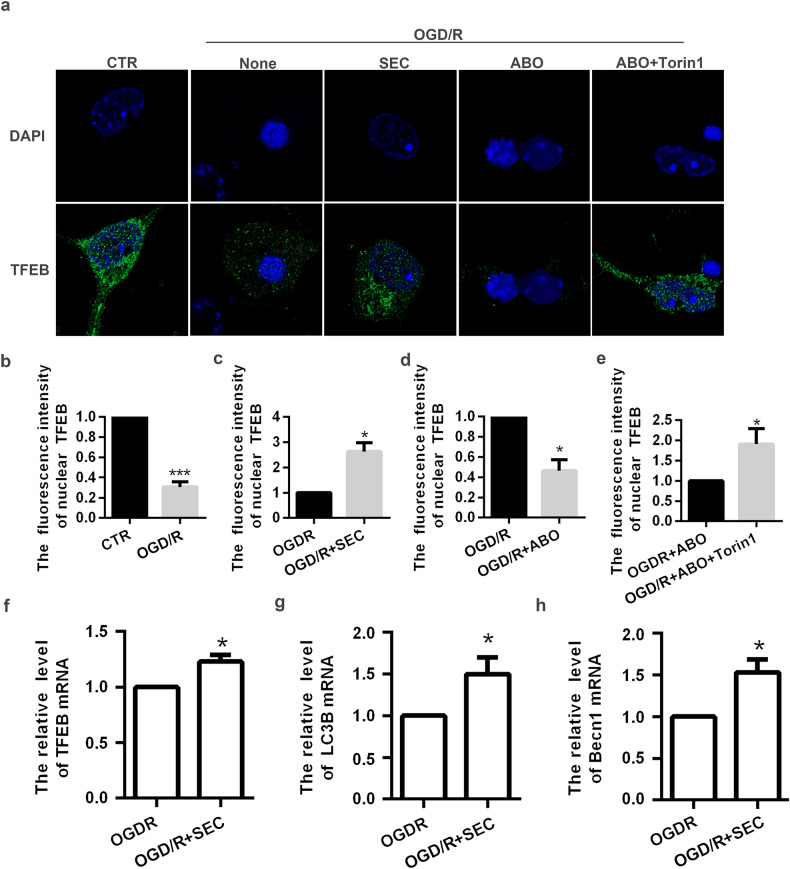
Activation of ANXA7 promotes autophagy through mTOR/TFEB. **a**–**e** Neurons were severally treated with DMSO (0.1%), SEC, ABO or ABO+torin1 for 1 h before OGD/R treatment, after which immunofluorescence was used to detect nuclear TFEB. Scale bars: 10 µm. **f**–**h** Neurons were severally treated with DMSO (0.1%) or SEC for 1 h before OGD/R treatment, after which qPCR was used to detect mRNA level of TFEB, LC3B and Becn1. Data are presented as means ± SD, * *P* < 0.05, ** *P* < 0.01, *** *P* < 0.001, *n* = 3.

Then, in order to explore SEC could promote autophagy through mTOR/TFEB signal pathway, we detect expression and distribution of TFEB by immunofluorescence staining. We found that activation of ANXA7 could increase expression and nuclear translocation of TFEB, whereas ANXA7 inhibition decreased that (Fig. 4a–d). Moreover, Torin1 can inhibit the effect of ABO and promote TFEB nuclear translocation (Fig. 4a, e). In addition, we detected the mRNA level of TFEB and autophagy genes downstream of TFEB after activating ANXA7, and found that ANXA7 activation can promote the transcriptional level of TFEB and downstream autophagy-related genes (Fig. 4f–h). The above results indicate that ANXA7 can promote autophagy by regulating mTOR/TFEB axis.

### ANXA7 interacts with LAMP5 to regulate spinal cord injury

**Fig. 5:**
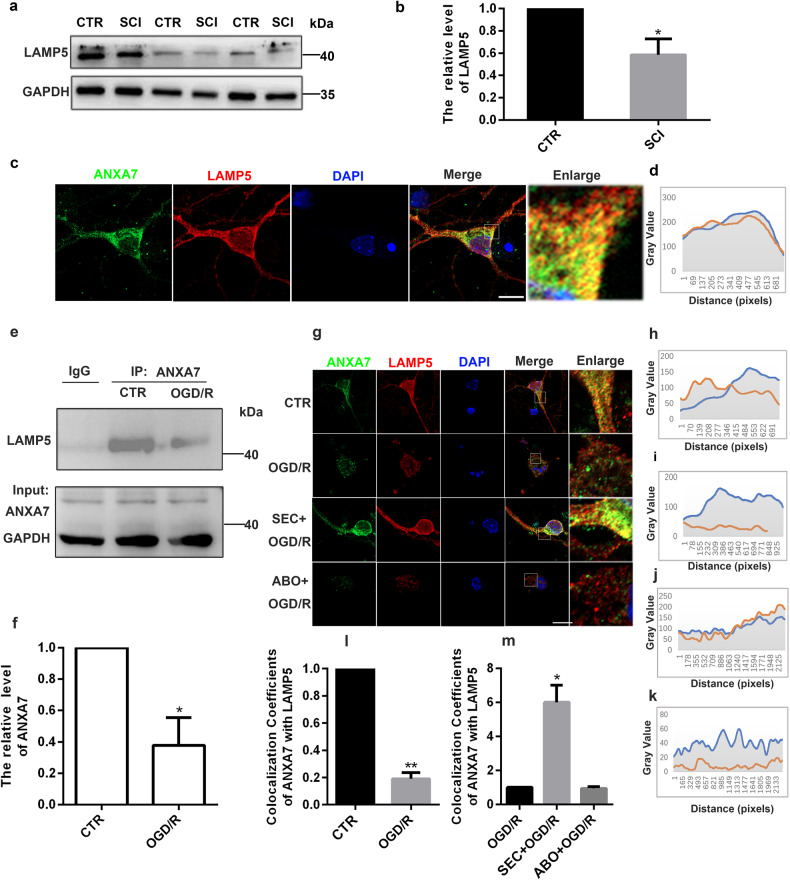
ANXA7 interacts with LAMP5 to stabilize its expression. **a**, **b** LAMP5 protein expression was detected in spinal cord 24 h after SCI. **c** Immunofluorescence was used to detect the co-localization between ANXA7 and LAMP5 in neurons. Scale bars: 10 µm. **d** ImageJ analysis the co-localization of ANXA7 and LAMP5. **e**, **f** Co-immunoprecipitation to detect the interaction between ANXA7 and LAMP5 after OGD/R treatment. **g** Immunofluorescence was used to detect LAMP5 expression and the co-localization between ANXA7 and LAMP5 in neurons after OGD/R, SEC + OGD/R and ABO + OGD/R treatment. Scale bars: 10 µm. **h**–**k** ImageJ analysis the co-localization of ANXA7 and LAMP5 in CTR, OGD/R, SEC + OGD/R and ABO + OGD/R treatment group. **l**, **m** Data analysis of Fig. 5g. Data are presented as means ± SD, * *P* < 0.05, ** *P* < 0.01, *n* = 3.

In the lysosomal-associated membrane protein family, member LAMP5 is brain and nerve specific. Previous studies have shown that ANXA7 could translocate to lysosomes, exerting the effect as inhibiting apoptosis and regulating autophagy. However, the regulatory mechanism is not clear. We validated the LAMP5 protein expression in spinal cord 24 h after SCI and found that LAMP5 protein level was significantly downregulated (Fig. 5a, b). Besides, immunofluorescence results showed that LAMP5 interacted with ANXA7 significantly in the cytoplasm of neurons (Fig. 5c, d). To figure out that there is an interaction between ANXA7 and LAMP5, immunoprecipitation was performed and the results showed that ANXA7 could directly interact with LAMP5 (Fig. 5e). Moreover, the interaction between LAMP5 and ANXA7 could be suppressed after OGD/R treatment (Fig. 5e, f). In addition, neurons were treated with SEC or ABO respectively, LAMP5 expression and co-localization between ANXA7 and LAMP5 were both detected. The results showed that SEC could increase the interaction between ANXA7 and LAMP5 and upregulate LAMP5 expression while ABO could inhibit this interaction and downregulate LAMP5 expression (Fig. 5g–m). These results indicated that ANXA7 activation could stabilize LAMP5 via increasing their interaction.

### Asp411 residue of ANXA7 is essential for maintaining the interaction between LAMP5 and ANXA7

**Fig. 6:**
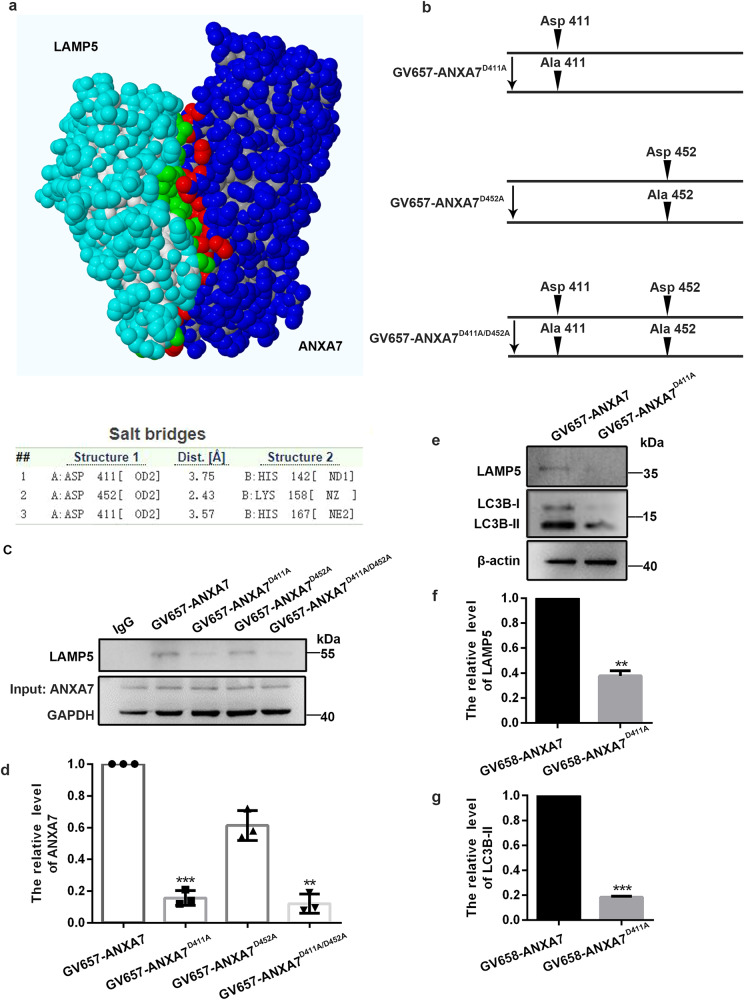
Asp411 residue of ANXA7 is an essential site for maintaining the interaction between ANXA7 and LAMP5. **a** Structure of interaction of ANXA7 and LAMP5, and the predictive results of amino acid sites regulating the interaction between ANXA7 and LAMP5. **b** Schematic representation of the mutation site in plasmid. **c**, **d** LAMP5 antibody was used in co-immunoprecipitation to detect the interaction between ANXA7 and LAMP5 after transfected with ANXA7 overexpression plasmid, ANXA7^D452A^ plasmid, ANXA7^D411A^ plasmid and ANXA7^D411A/D452A^ plasmid. **e**–**g** Western blot was used to detect protein level of LC3B and c -Caspase3 in neurons transfected with ANXA7 overexpression plasmid and ANXA7^D411A^ plasmid after OGD/R. Data are presented as means ± SD, * *P* < 0.05, ** *P* < 0.01, *** *P* < 0.001, *n* = 3.

To demonstrate the interaction between ANXA7 and LAMP5 and explore the amino acid sites of regulating their interaction, we queried the protein structure of ANXA7 and LAMP5 and predicted the potential amino acids sites that regulate the interaction by using Swiss-Model, Vasker lab and PDBePISA website. The results showed that Asp411 and Asp452 of ANXA7 are likely to be the binding sites of ANXA7 and LAMP5 (Fig. 6a). Based on website predictions, we constructed different ANXA7 mutant plasmids involving ANXA7^D411A^, ANXA7^D452A^ and ANXA7^D411A/D452A^ (Fig. 6b). Then we transfected them into 293 T cells and co-immunoprecipitation was performed in order to find out the binding site(s) between ANXA7 and LAMP5. The results showed that interaction of ANXA7 and LAMP5 was obviously decreased in groups transfecting with plasmid ANXA7^D411A^ or ANXA7^D411A/D452A^, while there’s no obvious change transfected with ANXA7^D452A^ only (Fig. 6c, d). Moreover, we detected the protein level of LAMP5 and LC3B-II, and they were decreased after transfection with ANXA7^D411A^ mutant plasmid (Fig. 6e–g). Above results proved that there is an interaction between ANXA7 and LAMP5, and the Asp411 residue of ANXA7 is the essential amino sites for maintaining the interaction and avoiding LAMP5 degradation further.

### LAMP5 increases lysosomal stability, promotes autophagy and inhibits apoptosis

**Fig. 7:**
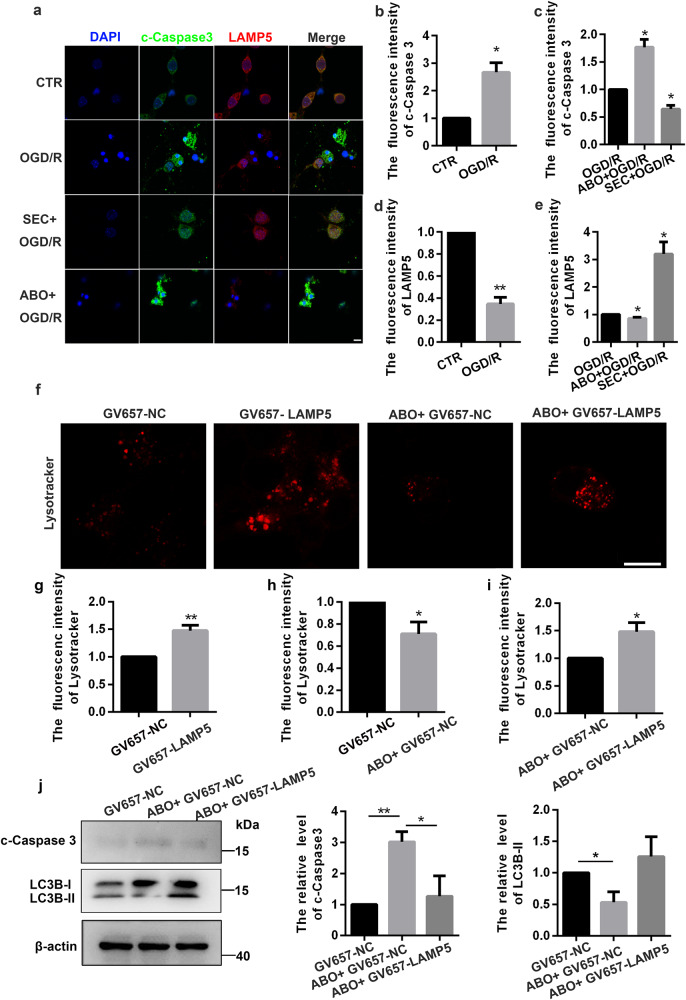
LAMP5 increases lysosomal stability, promotes autophagy and inhibits apoptosis. **a**–**e** Immunofluorescence was used to detect the protein level of c-Caspase3 and LAMP5 after OGD/R, ABO + OGD/R and SEC + OGD/R treatment. **f**–**i** Lyso-tracker DND99 was used to detect acidic environment of lysosomes in neurons transfected with negative control or LAMP5 overexpression plasmid, with or without ABO treatment for 1 h before OGD/R. Scale bars: 10 µm. **j** Neurons transfected with negative control or LAMP5 overexpression plasmid were treated with/without ABO for 1 h before OGD/R, and Western Blot was used to detect protein level of LC3B and c-Caspase3. Data are presented as means ± SD, * *P* < 0.05, ** *P* < 0.01, *n* = 3.

For demonstrating the regulatory effect of LAMP5 on neurons, we simultaneously detected the protein level of LAMP5 and c-Caspase3 after different treatment. The results showed that LAMP5 was significantly down-regulated and apoptosis was activated after OGD/R treatment, while SEC could rescue LAMP5 suppression and apoptosis induced by OGD/R (Fig. 7a–e). LAMP proteins family could regulate the barrier function of limiting membranes, and many channel forming proteins work together to control the flow of ions and metabolites. We speculated that LAMP5 had important regulatory effects on lysosomal stability. To verify the hypothesis, the Lysotracker was used to detect the stability of acidic environment in lysosomes. We found that transfection of GV657-LAMP5(LAMP5 over-expression) plasmid could increase the fluorescence intensity of lysosomal probes, which suggested that upregulation of LAMP5 is beneficial to stabilizing lysosomal function (Fig. 7f, g). In addition, upregulation of LAMP5 could not only rescue ABO induced instability of lysosomal acidity (Fig. 7f, h, i), but also promote autophagy and inhibit apoptosis compared to ABO-treated group (Fig. 7j). Hence, ANXA7 works with LAMP5 together to regulate neuronal autophagy and apoptosis.

### ANXA7 is able to promote the recovery of motor function in mice with spinal cord injury

**Fig. 8:**
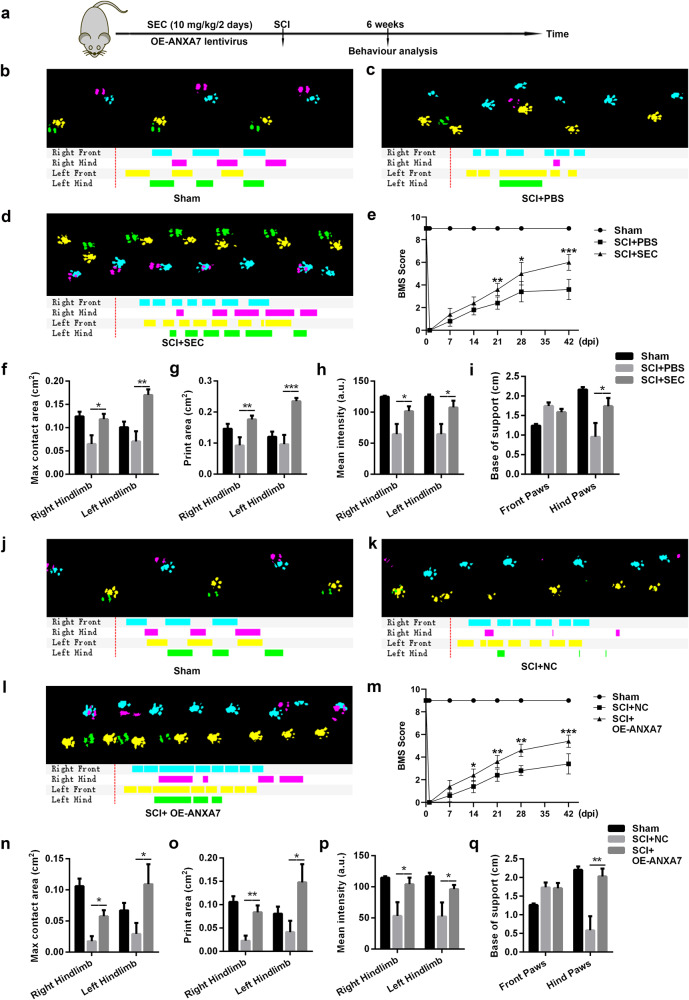
ANXA7 is beneficial to the recovery of motor function after spinal cord injury in mice. **a** C57BL/6 mice were used for establishing spinal cord injury models. SEC at a dose of 10 mg/kg/2d was injected by tail vein injection for three times before SCI treatment. OE-ANXA7 lentivirus was injected into spinal cord 3 days before SCI treatment. **b**–**d** Footprint images of mice in groups of sham, SCI + PBS and SCI + SEC. **e** BMS scores of sham, SCI + PBS and SCI + SEC groups. **f**–**i** Max contact area, Print area, Mean intensity and Base of support index of sham, SCI + PBS and SCI + SEC group. **j**–**l** Footprint images of mice in groups of sham, SCI + NC, and SCI + OE-ANXA7 group. **m** BMS scores of sham, SCI + NC, and SCI + OE-ANXA7 group. **n**–**q** Max contact area, Print area, Mean intensity and Base of support index of sham, SCI + NC, and SCI + OE-ANXA7 group. CatWalk analysis were conducted 6 weeks after spinal cord injury, while BMS score experiments were performed 1 day and every week after spinal cord injury in mice. BMS score is presented as means ± SD, while other data are presented as means ± SEM, * *P* < 0.05, ** *P* < 0.01, *** *P* < 0.001, *n* = 5.

To evaluate the regulatory effect of ANXA7 on spinal cord injury in mice, we injected ANXA7-overexpressing lentivirus and SEC separately into the C57BL/6 mice. 8-week-old C57BL/6 mice were used for spinal cord injury model establishment. The mice were divided into 6 groups with 5 mice each: sham group, SCI + PBS group and SCI + SEC group; sham group, SCI + NC group, and SCI + OE-ANXA7 group. After 6 weeks, CatWalk-XT system and BMS score were performed to assess the motor function of spinal cord injury mice (Fig. 8a). The results of the CatWalk assay showed that mice injected with SEC exhibited more uniform and stable footprint and better exercise ability (Fig. 8b–d, f–i). Moreover, the BMS score of the SEC treatment group was significantly higher than that of the PBS group (Fig. 8e). Similarly, the regularity and stability of footprints and the BMS score in the ANXA7 overexpression group were significantly higher than those in the negative control group (Fig. 8j–q). This suggested that both activation GTPase activity of ANXA7 and upregulation of ANXA7 could promote the recovery of motor function after spinal cord injury in mice.

### ANXA7 decreases neuron apoptosis and promotes the neural repair after spinal cord injury in mice

**Fig. 9:**
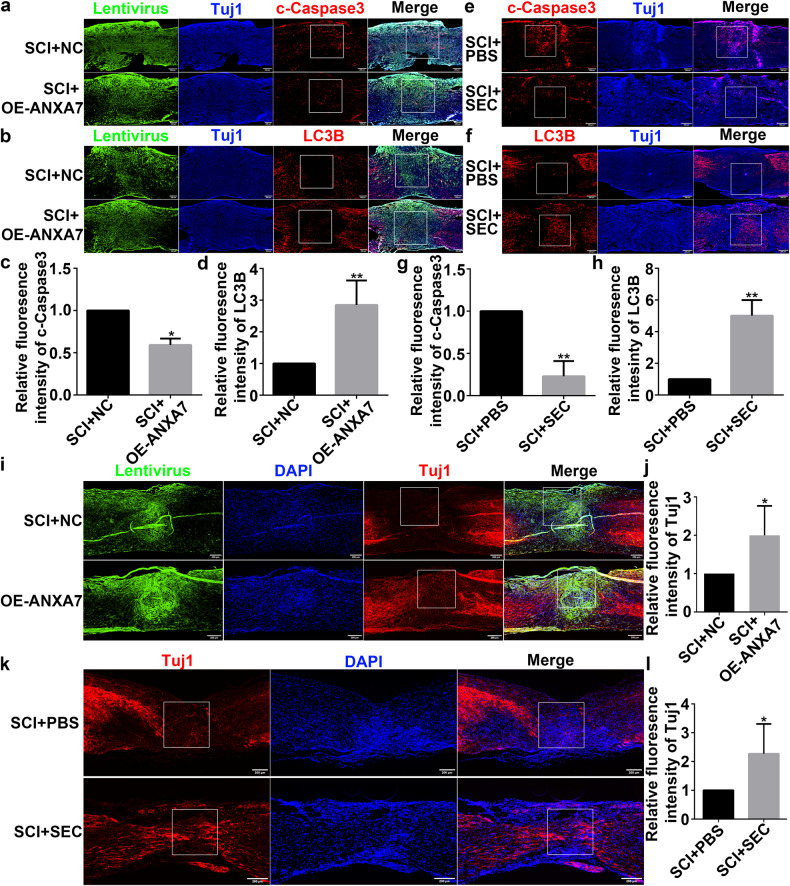
ANXA7 promotes neural repair after spinal cord injury in mice. **a**–**d** Immunofluorescence staining and quantification of c-Caspase3 and LC3B in SCI + NC (injecting unloaded lentivirus) group and SCI + OE-ANXA7 group after 3 days of SCI. **e**–**h** Immunofluorescence staining and quantification of c-Caspase3 and LC3B in SCI + PBS group and SCI + SEC group after 3 days of SCI. **i, j** Immunofluorescence staining and quantification of Tuj1 in SCI + NC group and SCI + OE-ANXA7 group after 6 weeks of SCI. **k**, **l** Immunofluorescence staining and quantification of Tuj1 in SCI + PBS group and SCI + SEC group after 6 weeks of SCI. Data are presented as means ± SD, * *P* < 0.05, ** *P* < 0.01. Scale bars: 200 µm.

Based on above results, we used animal models to demonstrate ANXA7 upregulation or activation could both promote neural repair by inhibiting apoptosis via activating autophagy after spinal cord injury in vivo. Immunohistochemistry staining results demonstrated that ANXA7 upregulation could promote the expression of LC3B and inhibit the expression of c-Caspase3 three days after spinal cord injury in mice (Fig. 9a–d). Meanwhile, activation GTPase activity of ANXA7 has similar effect in mice (Fig. 9e–h). Therefore, targeting ANXA7 could promote autophagy and thus inhibit apoptosis in neuron. We then examined tuj1 expression to verify the repair effects of ANXA7 after spinal cord injury in vivo. The results showed that upregulation and activation GTPase activity of ANXA7 could both promote neural repair after spinal cord injury (Fig. 9i–l). Thus, it could be demonstrated that ANXA7 could decrease neuron apoptosis via activating autophagy and promote the neural repair after SCI in vivo.

## Discussion

Spinal cord injury causes impairment of neurological functions involving motor and sensory functions, and even leads to temporary or permanent disability in patients [35]. However, few patients suffer from spinal cord injury could get neurological function recovery due to the lack of definite and effective treatment strategies [36]. Therefore, it has been the focus to explore the molecular mechanism of neuronal damage after spinal cord injury and to propose corresponding solutions in research field related to spinal cord injury. Current studies on spinal cord injury mainly focus on regulating neuronal death, inhibiting the activation of inflammatory cells, inhibiting the glial scar caused by astrocytes, promoting the differentiation of other cells into neurons and exosome injection [37**–**40]. Stem cell transplantation was performed to improve the injury microenvironment and material engineering was applied for spinal cord injury repair [4, 41]. However, it is still necessary to explore the new mechanisms and put up effective treatment for spinal cord injury. This study focuses on the exploration of new and specific targets and mechanisms for regulating neural repair after spinal cord injury.

ANXA7 is a special annexin with GTPase activity. It can not only regulate the interaction of organelles mediated by membrane fusion, but also regulate various physiological processes such as autophagy and apoptosis [42, 43]. Interestingly, we observed the specificity of the ANXA7 distribution in spinal cord neurons. It is abundant in neurons and few in other type cells, which predicts that it may have specific regulatory role in neuron. And with the deepening of spinal cord injury, the expression level of ANXA7 gradually decreased. Our data demonstrated that ANXA7 is significantly downregulated spinal cord tissues of mouse after injury as well as neuron after OGD/R. When we used SEC to activate ANXA7, it can promote autophagy and inhibit apoptosis of injured neuron.

Translocation of ANXA7 to the lysosome inhibits apoptosis, but the mechanism is unclear. Our study found that the lysosome-associated membrane protein LAMP5 is significantly co-localized with ANXA7. The expression of the two interacting proteins decreases after spinal cord injury, and the interaction decreases also. Activation of ANXA7 can promote the interaction, and increase neuronal autophagy but inhibit apoptosis, which indicates that enhancing the interaction between ANXA7 and LAMP5 has a protective effect on neurons. Subsequently, we observed that the Asp411 amino site of ANXA7 is a key binding site for regulating interaction of ANXA7 and LAMP5, and the mutation of Asp411 could suppress the interaction.

The mTOR signaling pathway plays an important role in multiple cellular functions, such as cell metabolism, autophagy and survival [44**–**46]. A few studies have shown that mTOR regulates both neuroprotective and neural regenerative functions in trauma and various diseases in the central nervous system (CNS) [47, 48]. Studies have shown that mTOR inhibition could reduce neural tissue damage and locomotor impairment after spinal cord injury in mice [27, 29, 30]. Since mTOR plays an important role in regulating various physiological processes, we guess if it takes part in regulation of neuron after spinal cord injury. Our results demonstrated that activation of GTPase activity of ANXA7 could inhibit phosphorylation of mTOR, thus promoting autophagy. Furthermore, SEC can promote the nuclear translocation of TFEB, thereby promoting the expression of downstream autophagy related genes to enhance autophagy. These findings indicate that we identified a novel factor that regulate the mTOR/TFEB pathway. But the way in which ANXA7 inhibits phosphorylation of mTOR and whether other mechanisms that activating ANXA7 can also protect neurons through remain need to be studied. Could ANXA7 directly regulates mTOR activity and whether it mediates interaction between organelles by regulating organelle membranes fusion will be our next research emphasis.

In this study, we validated the mechanism that activation of ANXA7 has a protective effect on injured spinal cord. To elucidate the application prospect of ANXA7 as a specific target for spinal cord injury repair, we used mice spinal cord injury model to verify the significant repairing effect of targeting ANXA7 on spinal cord injury. ANXA7 upregulation and activation can inhibit the apoptosis of neurons, promote the repair of spinal cord injury, and improve the motor function in mice after spinal cord injury.

As a traumatic disease, unlike cancer and cardiovascular disease, spinal cord injury has no obviously significant genetic characteristics, and its onset is often accompanied by a large release of inflammatory factors and activation of oxidative stress pathways [49]. Therefore, the treatment of spinal cord injury lacks specific targets and corresponding repair strategies. It plays a crucial role to find specific targets in regulating spinal cord injury. Our study proved that ANXA7 is a specific target for regulating neuronal repair after spinal cord injury, and activation of ANXA7 can promote the functional repair of injured neurons by itself, rather than relying on materials and other external auxiliary measures. This is of great significance to patients suffered from spinal cord injury, and also hints at the prospect of clinical application of SEC, the activator of ANXA7.

## Conclusions

**Fig. 10:**
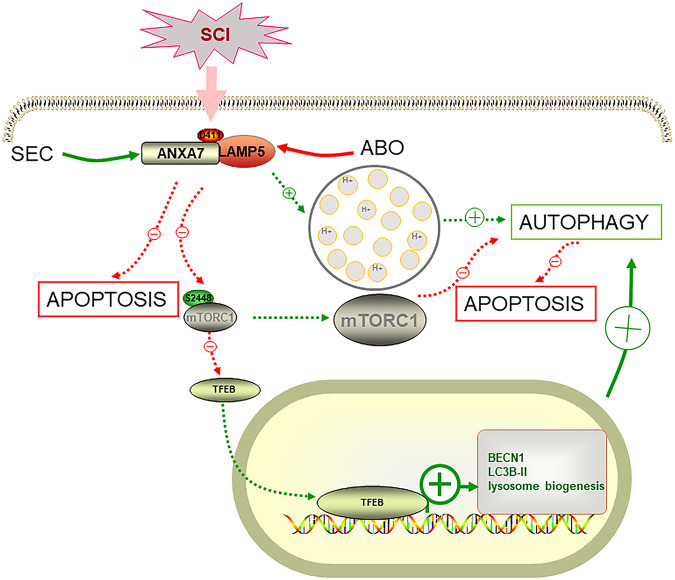
Diagrammatic sketch for mechanism that ANXA7 regulates neuron apoptosis after spinal cord injury. ANXA7 could interact with LAMP5 through the binding site ANXA7^D411^ to regulate autophagy and apoptosis. Interaction between ANXA7 and LAMP5 is significantly inhibited after spinal cord injury. SEC, the activator of ANXA7, can promote the interaction between ANXA7 and LAMP5 by activating the activity of ANXA7, thereby promoting autophagy by stabilizing the acidic environment of lysosomes and regulating mTOR/TFEB signaling pathway, thus inhibiting apoptosis, repairing damaged neurons, and promoting the repair of spinal cord injury. Red arrow and symbol ‘−’: inhibiting effect; Green arrow and symbol ‘+’: promoting effect.

In summary, we discovered that ANXA7 could interact with LAMP5 through ANXA7^D411^ to regulate autophagy and apoptosis and the interaction between ANXA7 and LAMP5 is significantly inhibited after spinal cord injury. SEC, the activator of ANXA7, can promote the interaction between ANXA7 and LAMP5 by activating the activity of ANXA7, thereby promoting autophagy by stabilizing the acidic environment of lysosomes and regulating mTOR/TFEB signaling pathway, thus inhibiting apoptosis, repairing damaged neurons, and promoting the repair of spinal cord injury (Fig. 10). Furthermore, upregulation or activation of ANXA7 could both promote autophagy but inhibit neuronal apoptosis, and further promote motor recovery in mice after spinal cord injury. Hence, our study finds a new mechanism for the regulation of autophagy by ANXA7, and also puts up a new specific target for spinal cord injury repair.

## Materials and methods

### Cell culture and treatments

In this study, primary neurons were isolated from the cerebral cortex of E17–19 days C57BL/6J embryos. The cerebral cortex was dissected after vascular membrane removing and washed with high-glucose DMEM (BasalMedia, Cat# L110KJ) at 4 °C. The cerebral cortex was cut into about 1 mm^3^ pieces. The tissues were collected after centrifuging at 1200 rpm for 5 min and digested with 2 mg/mL papain (Worthington, Cat# LS003126) and DNAase (Sigma, Cat#D5025) solution for 15 min at 37 °C, and then filtrated through a 40 μM strainer to get single cells. Single cells were collected and resuspended with high-glucose DMEM containing 10% fetal bovine serum (CellMax, Cat# SA101.02) and 1% penicillin/streptomycin (Gibco, Cat# 15140122), and then, cells were cultured on plates or dishes precoated with poly-l-lysine (Sigma-Aldrich, Cat# P4707). For 6-well plate, 2 million cells were used each well, and 6 million cells for 6 cm dishes, 12 million cells for 9 cm dishes and 1.5 × 10^5^ cells for glass-bottom dishes. The medium was replaced with neurobasal medium (Gibco, Cat# 21103049) containing 2 mmol/L glutamine (Gibco, Cat# 25030081), 1% B27 Supplement (Gibco, Cat# 17504044) and 1% P/S after 4 h. The medium was changed every 3 days.

### Oxygen–glucose deprivation/reoxygenation treatment

The medium was replaced by glucose-free DMEM (BasalMedia, L160KJ) after washing for three times and neurons were placed in hypoxic conditions (37 °C, 94% N_2_, 1% O_2_, and 5% CO_2_) for 1 h. Then, the medium was changed to normal neuronal culture medium and neurons were incubated under normal incubatory conditions for 3 h.

### Plasmid transfection

The coding region of human ANXA7 and LAMP5 was subcloned into GV657 expression vector to produce GV657 -ANXA7 and GV657 -LAMP5. Similarly, D411A mutant, D452A mutant and D411A mutant plus D452A mutant (Asp411 and Asp452 of ANXA7 were mutated to Alanine, respectively) were subcloned into GV657 expression vector. All the constructs were confirmed by DNA sequencing. Cells were plated onto 6 cm dish (NEST Biotechnology, Wuxi, China) at a density 1 × 10^6^/mL and grew for 24 h. When cell density reached 70–80%, cells were transfected with indicated expression vectors using Lipofectamine 3000 reagent (Invitrogen, L3000150) according to manufacturer’s protocols. Finally, cells were harvested and protein expression was analyzed by Co-IP and Western Blotting.

### Co-immunoprecipitation

Cells were lysed by using Western and IP lysing buffer (Beyotime Biotechnology, Cat# P0013) and the bicinchoninic acid protein assay kit (Beyotime Biotechnology, Cat# P0010) was used for protein concentrations quantification. The total amount of more than 500 μg protein content of supernatant was incubated with specific antibodies or normal corresponding IgG at 4 °C overnight, then pre-cleared protein A/G agarose beads (Beyotime Biotechnology, Cat# P2012) were added into the supernatant and incubated at 4 °C for 3 h. Finally, the beads were collected, washed for three times and eluted with 2× SDS loading buffer to release proteins. The immune precipitated protein was detected by Western Blotting assay.

### Western blotting

Cells were collected and lysed by RIPA buffer (Beyotime Biotechnology, Cat# P0013B) with protease inhibitor cocktail (20×). For further lysing, ultrasonication was performed. Then, cells were centrifuged with 12,000 rpm at 4 °C for 25 min. The supernatant was collected and boiled with loading buffer (5×) for 10 min. Proteins were separated by SDS-PAGE electrophoresis, and electrotransferred onto the PVDF membrane (Millipore, Cat# ISEQ0010, IPVH0010). Then, the membrane was blocked with 5% Bovine Serum Albumin (BSA, Solarbio Life Sciences, Cat# A8020) for 1 h and incubated with primary antibodies as follows at 4 °C overnight: Annexin A7 (Proteintech, Cat# 10154-2-AP), LAMP5 (Santa Cruz, Cat# sc-398190), LC3B (Abcam, Cat# ab192890), mTOR (Proteintech, Cat# 66888-1-Ig), p-mTOR (Proteintech Cat# 67778-1-Ig), GAPDH (CST, Cat# 5174 S), beta-actin (CST, Cat# 4970 S), Cleaved-caspase3 (CST, Cat# 9661 S). After overnight incubation, the membrane was washed with TBST for three times (5 min per time) and then incubated with horseradish peroxidase-conjugated Rabbit or Mouse secondary antibodies (1:15,000; Zhongshan Golden Bridge Biotechnology, Beijing, China). Protein bands were visualized by using electrochemiluminescence reagent.

### mRNA extraction and quantification

Cells were lysed by TRIzol (Invitrogen, Carlsbad, CA, US) and total RNA was collected by using the Total RNA Extraction Kit (Solarbio Life Sciences, Cat# R1200). RNA was reversely transcribed to cDNA by using Revert Aid First Strand cDNA Synthesis Kit (Thermo Fisher Scientific, Waltham, MA, US). The quantitation of mRNAs was detected by using quantitative real-time PCR (RT-qPCR) with Pro Taq HS SYBR Green (Accurate Biotechnology (Hunan) Co.,Ltd, ChangSha, China, Cat# AG11701). All values were normalized to an endogenous glyceraldehyde-3-phosphate dehydrogenase (GADPH) control. The primers used were as follows: ANXA7 F:5′- TTCTTTGCTGAACGACTCTACT-3′, R:5′- GAACAAGGTCAATCTCACTTCG −3′; TFEB F: 5′-CCTCCTCCTCCTCCTCCTCCTC-3′, R:5′- CTATTGCCCTCTTCTGGTGTGTCTG −3′; LC3B F: 5′- GCTGGCACTTGGGAGGACATTG-3′, R: 5′- CCTGCTGACAACACTGCTGGAC −3′; Becn1 F: 5′- CGCAAGGTGGTGGCAGAGAAC −3′, R: 5′- GAAGGCAGAAGAGCAGGGCAAG −3′; GAPDH F: 5′- TGTCTCCTGCGACTTCAACA −3′, R: 5′- GGTGGTCCAGGGTTTCTTACT −3′.

### Immunofluorescence assay

Cells were seed on glass bottom dishes (SORFA Life Science, Beijing, China) and fixed with 4% paraformaldehyde (w/v) after treatment at room temperature for 30 min. Then the cells were permeabilized with 0.1% Triton X-100 for 10 min after washing with PBS for three times. The samples were blocked with 5 % BSA at room temperature for 30 min and incubated with primary antibodies at 4 °C overnight: Olig2 (Abcam, Cat# ab109186), Iba1(Abcam, Cat# ab178846), GFAP (Abcam, Cat# ab68428), NeuN (Abcam, Cat# ab177487), LAMP2 (Proteintech, Cat# 66301-1-Ig), TFEB (Proteintech, Cat# 13372-1-AP), Annexin A7 (Proteintech, Cat# 10154-2-AP), LAMP5 (Santa Cruz, Cat# sc-398190), LC3B (Abcam, Cat# ab192890), Cleaved-caspase3 (CST, Cat# 9661 S). Samples were incubated with secondary antibodies at 37 °C for 1 h after washing with TBST for three times. Finally, samples were stained with DAPI for 10 min to show nucleus, and fluorescence signals were detected by laser scanning confocal microscopy (Zeiss LSM900, Germany).

### Immunohistochemistry

The mice were perfused with cold PBS and followed by 4% paraformaldehyde under anesthesia. About 1.0 cm spinal cord segments involving injury area were used for following experiments. Tissues were fixed in 4% paraformaldehyde solution at 4 °C overnight and dehydrated in sucrose solution with increasing concentration (10%, 20%, 30%). Then the tissues were embedded with TissueTek OCT compound (SAKURA, Cat# 4583, Torrance, CA, USA) and sliced into pieces with 10 μm thickness. The pieces were permeabilized and blocked by using 5% BSA containing 0.1% Triton X-100 and incubated with primary antibodies overnight. Rabbit or mouse secondary antibodies labeled with specific fluorescence were used for staining at room temperature for 1 h. The nucleus was stained by DAPI. For visualization, images were acquired through Zeiss LSM 900 confocal laser microscope.

### Spinal cord contusion injury model establishment

All experiments were authorized by the Ethics Committee of Qilu Hospital of Shandong University, and the procedures were carried out in adherence to the Animal Research Ethics Committee of the Qilu Hospital of Shandong University (Issue NO. DWLL-2022-087). 8 weeks old female C57BL/6 J mice (*n* = 5 per group) were used for preparing spinal cord contusion injury model. The allocation of mice in each group were randomized and blinded. 5% isoflurane in 2 L/min oxygen for induction of anesthesia. For maintenance of anesthesia, 1% isoflurane in 1 L/min oxygen. The back fur was shaved to expose the surgical area which was sterilized with povidone iodine antiseptic liquid. A vertical midline incision at the T10 vertebral level was made by using a surgical scalpel and the skin was reflected. Blunt dissection was performed to separate subcutaneous tissue and fascia and to expose spinous processes. And then, para-spinous muscles were separated to expose the laminae. The laminae were opened bilaterally to expose spinal cord. The spinal cord was stroke medially by Precision Strike (RWD Life Technology, Shenzhen, China) with parameters as: diameter, 1.0 mm; velocity, 1.5 m/s; duration, 0.5 s; depth, 0.5 mm. Then, muscles, subcutaneous tissue, fascia and skin were closed. For post-operative care, antibiotics were used for the seven days and the bladder was emptied manually at least twice daily.

### Locomotor function investigation

Hind limb motor functions of the mice were evaluated one month after the surgery. Animals were allowed to walk freely in an open field, and BMS locomotion testing was conducted by blinded observations within 5 min for each animal.

### CatWalk gait analysis

The CatWalk walking track test (Noldus Inc., Wageningen, Netherlands) consists of an illuminated-walkway glass floor with a high-speed video camera equipped with a wide-angle lens. The paws prints were automatically recorded by CatWalkTM XT 10.6 software as the animal crossed the pathway. Gait analysis was performed 6 weeks after spinal cord injury model preparation. Each mouse performed three runs during each analysis period.

### Statistical analysis

For the results, the difference between two groups was evaluated by Student’s t-test. For comparisons among more than two groups, one-way analysis of variance (one-way ANOVA) was used for analysis. Statistical software SPSS 20.0 was used for statistical analysis.

### Supplementary information

SUPPLEMENTAL MATERIAL

## Data Availability

All data generated or analyzed during this study are included in this published article.
