# Supplementary figures and images for: Targeting ANXA7/LAMP5-mTOR axis attenuates spinal cord injury by inhibiting neuronal apoptosis via enhancing autophagy in mice
Source: Cell Death Discov. 2023 Aug 24;9:309. doi: 10.1038/s41420-023-01612-w (PMC10449888; doi:10.1038/s41420-023-01612-w)

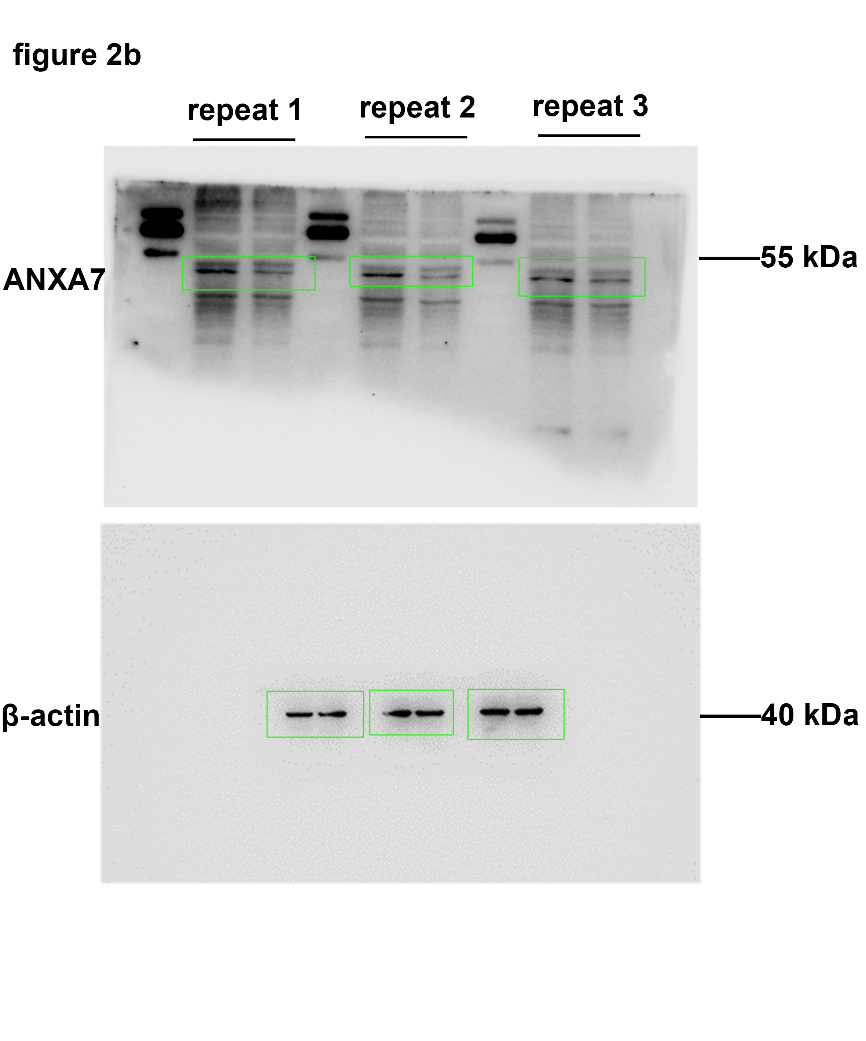


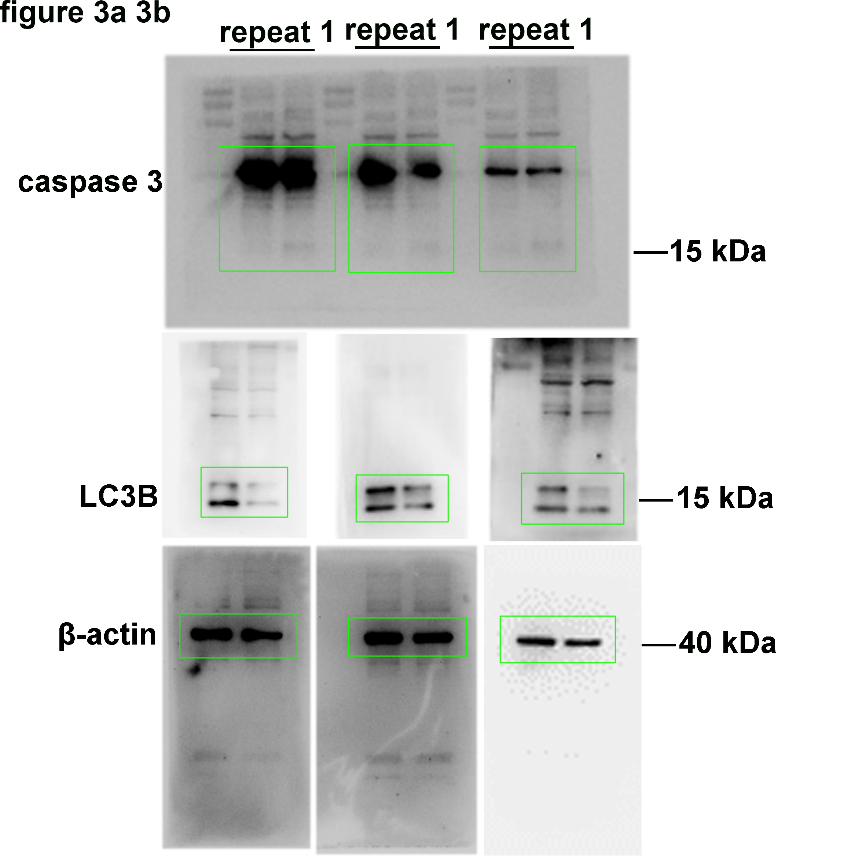


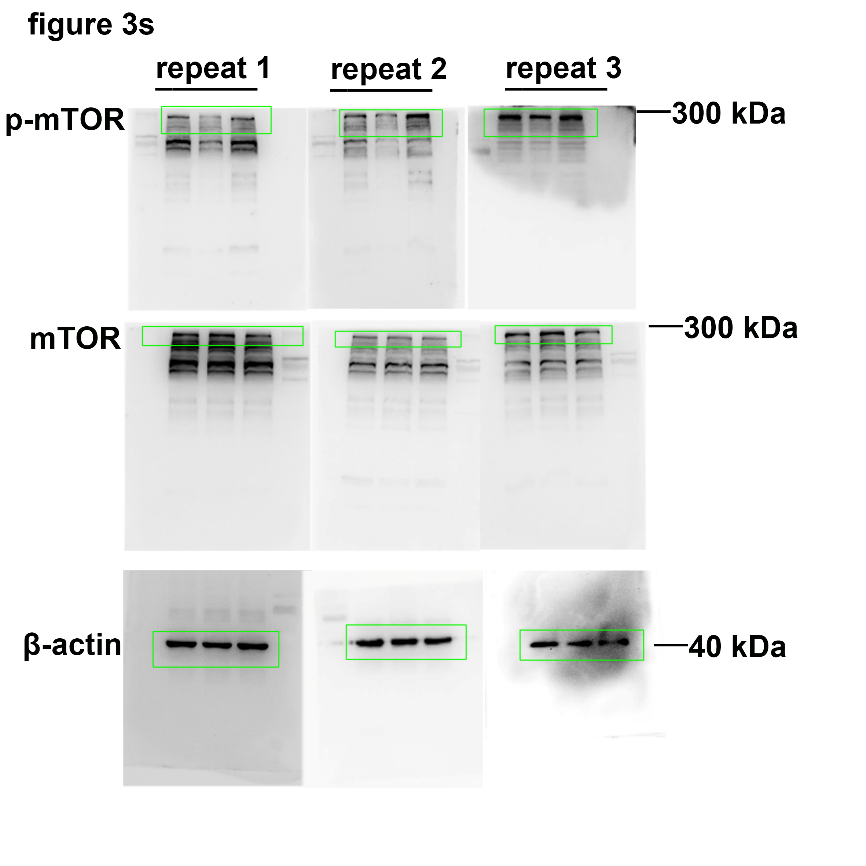


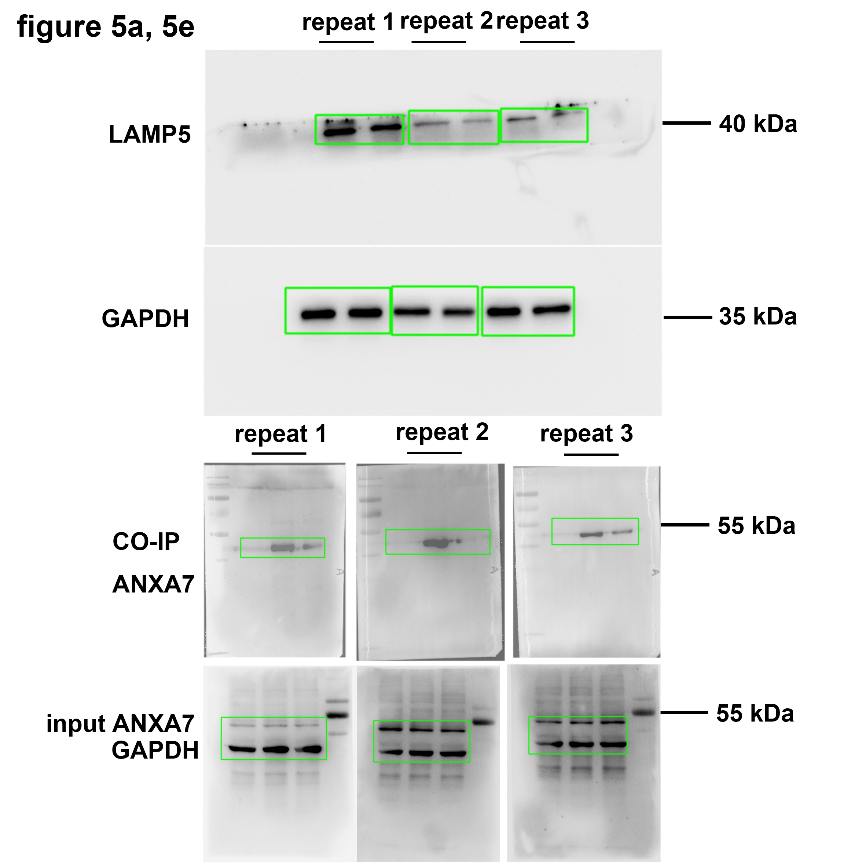


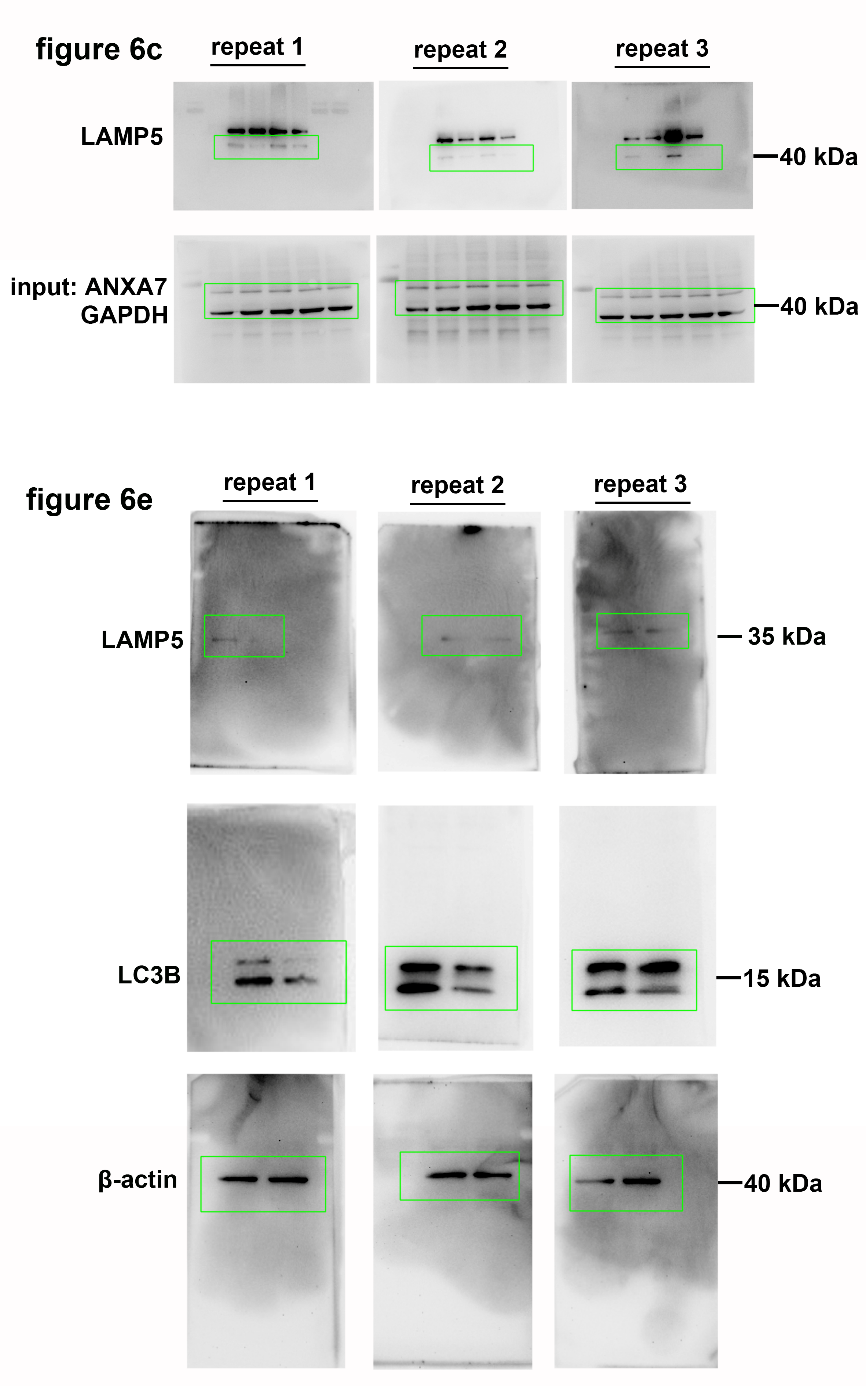


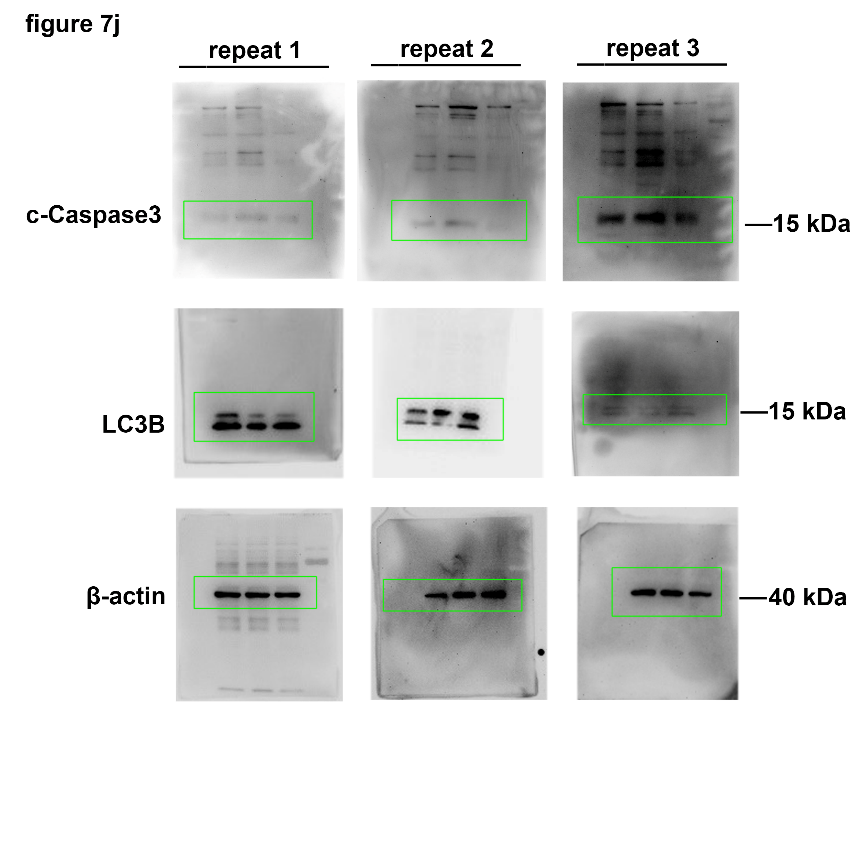

Supplement: Supplementary file 1 — SUPPLEMENTAL MATERIAL [file 41420_2023_1612_MOESM1_ESM.docx]
